# Postpartum depression and life experiences of mothers with an immigrant background living in the south of Sweden

**DOI:** 10.1080/17482631.2023.2187333

**Published:** 2023-03-07

**Authors:** Maude Johansson, Kajsa Ledung Higgins, Leoine Dapi Nzefa, Ylva Benderix

**Affiliations:** Department of Psychology, Linnaeus University, Växjö, Sweden

**Keywords:** Depression, integration, health literacy, life experiences, migrated mothers, postpartum depression: psychosomatic symptoms, qualitative study

## Abstract

**Purpose:**

Postpartum Depression (PPD) —a common health problem for mothers’ postpartum increases the risk of negative interaction between mothers and infants as it reduces the former’s ability to respond to the latter’s needs appropriately. Migrant mothers exhibit a higher prevalence of risk factors for PPD. Hence, this study aimed to investigate migrant mothers’ life experiences pertaining to motherhood and PPD.

**Methods:**

Qualitative interviews were conducted with 10 immigrant mothers in the south of Sweden during 2021.

**Results:**

The qualitative content analysis revealed the following main themes: 1) PPD (two sub themes—psychosomatic symptoms and burden of responsibility due to feelings of loneliness); 2) mistrust of social services (one sub-theme—afraid of losing their children and Swedish social services’ lack of understanding); 3) inadequate healthcare (two sub-themes—limited healthcare literacy for migrant mothers and language barrier; 4) women’s coping strategy for well-being (two sub-themes—better awareness and understanding of the Swedish system and society, and freedom and independence in the new country).

**Conclusions:**

PPD, mistrust of social services, and inadequate healthcare lacking personal continuity were common among immigrant women, thus precipitating discrimination—including lack of access to services because of limited health literacy, cultural differences, language barriers, and insufficient support.

## Introduction

Postpartum Depression (PPD) is a common health problem for mothers during pregnancy and after childbirth. Becoming a parent is associated with an increased risk of developing depressive symptoms in women and it affects approximately 10–15% of women (Brummelte & Galea, 2016; Halbreich & Karkun, 2006). Depression and PPD not only affects mothers, but also increases the risk of negative interaction between mothers and infants as the depression reduces her ability to respond to the child’s needs in a proper way. The interaction between mother and child plays a key role in the latter’s emotional and cognitive development.

PPD is a health problem affecting mothers from all countries and cultures (Halbreich & Karkun, 2006). In general, migrant women are at higher risk of depression (rates of 0–60% have been reported among migrant women (Alhasanat & Fry McComish, 2015; Halbreich & Karkun, 2006)) compared with 10–15% among non-migrant women (Brummelte & Galea, 2016; Halbreich & Karkun, 2006).

Migrant mothers have a higher prevalence of risk factors such as previous depression, poor socioeconomic circumstances, complicated pregnancy, labour and birth. They also often have a pre-migration history of trauma, abuse and stressful life events, and worries about relatives left behind (Alhasanat & Fry McComish, 2015; Shakeel et al., 2018; Tobin et al., 2015).

A meta-ethnographic study contains experiences of immigrant women having symptoms of PPD when living in a high-income country. The women described that language was a significant obstacle, and that their poor knowledge in the mainstream language hindered them from talking about their experiences and expressing emotions which affected receiving help during birth experiences. Women reported loneliness, being irritated, angry, exhausted, emotionally overwhelmed, and crying easily. Some witnessed a loss of identity compared to what they enjoyed in their country of origin. Several women came from countries where motherhood was of greater status than in the country they live in now. In the new country they were scared of being looked upon as a bad mother, and this started thoughts that their baby could be taken away from them (Schmied et al., 2017). Numerous women ignored their feelings of non-well-being and kept on living as normally as possible. They did not consider their difficult emotions as something alarming because postnatal depression is not recognized in their country of origin and could be looked upon as having mental problems. Also, women realized their stress and related it to be a side effect of being a migrant, living in a new country with less friends, social networks and family support (Dennis & Chung Lee, 2006; Schmied et al., 2017). A qualitative systematic review found that the mothers’ inability to disclose their feelings was a common help-seeking barrier. The behaviour was often reinforced by their family and the healthcare reluctance to respond to the emotional and practical needs of the mothers. The mothers’ lack of knowledge about PPD was also an obstacle for help-seeking and made the mothers unable to recognize the symptoms of depression (Dennis & Chung Lee, 2006; Schmied, Virginia et al., 2017).

The Swedish National Board of Health and Welfare recommended in 2010 that all mothers should be invited to participate in a routine screening for PPD 6–8 weeks after giving birth ((Berlin et al., 2006; Skoog et al., 2017, 2022). The intervention of routine screening is important to identify mothers with PPD and give them treatment. The immigrant mothers’ routine screening of PPD seemed to be a great challenge for healthcare with risk for not being screened (Berlin et al., 2006; Skoog et al., 2017, 2022). A previous Swedish prevalence report of PPD showed that few migrant mothers participated in the study (Johansson et al., 2017). In Sweden, studies on refugees and migrant mothers are few despite Sweden being one of the most multi-racial countries in Europe. Studies focused on nurses working with mothers in child healthcare found deficiencies in their working conditions and cultural competence and this may be a reason why immigrant women seemed to have limited access to screening for PPD compared to resident women (Berlin et al., 2006).

Screening for PPD in migrant women is important and needs to be developed and supported by the healthcare as they are especially vulnerable due to lack of social support, poverty, language barriers, lack of knowledge about the new health system and how to access care (Tobin et al., 2015).

More research is needed to explore migrant mothers’ life experiences of being a mother in Sweden with special focus on the mothers’ experiences of PPD. Hence, this study aimed to investigate migrant mothers’ life experiences of motherhood and PPD.

## Materials and methods

### Participants and settings

This qualitative study was conducted in a region of Sweden and included women with immigrant backgrounds having residence permits and who gave birth in their country of origin and/or in Sweden. Three information meetings were conducted, one meeting within the Integration Project, one meeting at the Family Centre, and one meeting with the Parental Group. From these three meetings 10 mothers provided consent to participate in the study, first by email and, thereafter, before the interview, they also provided signed consent. All 10 mothers were interviewed by the first author.

### Data collection

A semi-structured interview guide was used with open questions based on a framework, addressing aspects of the women’s experience of being parent, mental health, integration in Sweden, pregnancy and delivery experiences.

The participants provided information about their experiences of being immigrant mothers in Sweden and that of PPD, depressive symptoms, and parental stress when asked retrospective questions during the in-depth interviews. They were interviewed until data saturation, when both the participants and researchers felt that no more information could be obtained.

The interviews were conducted in Swedish or in English and took about one and one and half hours.

### Characteristics of participating mothers

The average age for the mothers were 30 (25–40) years. The numbers of children were 1–6. Four women had primary school education (3 years of school from their home countries). One had secondary school education, five of the mothers had university education, four of them were educated in Sweden, one was educated in another country. Only one mother had work experience (now on parental leave). Five mothers were unemployed and five were on parental leave. Eight women were married or cohabiting, and two mothers were divorced. These women were born in different countries such as Africa, East Asia, Latin America and East Europe (see Table I).

**Table I.** Characteristics of participating mothers (*N* = 10).

Characteristics		Number
Age		30 (25–40)
Marital status	Married/co-habituating	8
	Divorced	2
Age of marriage^1^		16 (16–25)
Number of children^1^		3 (1–6)
Education	Primary school	4
	Secondary school	1
	University	5
Occupation	No-job	6
	Parental leave	4

^1^Mean (Min—Max).

### Ethical considerations

The study received ethical approval from the Regional Ethics Review Board in Linköping, Sweden (Register number 2019/06439), and was performed in accordance with the Helsinki Declaration. All research procedures were conducted in accordance with the requirement of the Regional Ethics Review Board in Linköping, Sweden, including the informed consent of participants. Written informed consent was obtained from all women before the study. All information collected from participants was kept confidential, with no trace of identification details in the final report.

### Analysis

Interviews were audio-taped, transcribed verbatim. The interviews were analysed using qualitative content analysis (Graneheim & Lundman, 2004). The 10 interviews repeatedly were read for us to become accustomed to—and receive an overview of—the interview material. We repeatedly read the entire text. After familiarizing ourselves with the material, we began the coding process. We used mean units, a word or a sentence that answered interview questions. Authors underlined the texts and took notes of recurring meaning-bearing phrases consisting of keywords that described a series of similarities that all authors considered were related to each other and to the questions for the study to see similarities and differences in empiricism. Sentences, phrases or words that contained information relevant to the questions were picked and the surrounding text included to retain the context. The meanings units were compared to determine differences and similarities and were grouped into categories. These categories were a description of the manifest content. We then abstracted themes and sub-themes from the descriptions and interpretations. Sub-themes were threads of meaning running through the condensed text and an interpretation of their latent content This process included several back-and-forth steps to find the meaning-bearing phrases and categories that really reflected the mothers’ meaning in the interviews (Graneheim & Lundman, 2004). In our material it was helpful to divide the categories into subcategories (Graneheim & Lundman, 2004). Finally, categories were grouped into four themes.

An open-minded approach and a well-prepared interview guide were used to contain preconceptions, and to explore and obtain new knowledge. The trustworthiness of the analytical process was strengthened by the joint work of the authors, whose repeated discussions about different cultural backgrounds improved reflexivity throughout the research process.

This study comprised four researchers with different backgrounds. The first author was a psychologist and psychotherapist with a long experience in working with parents having PPD. The author also conducted research in the areas of PPD and parental stress among Swedish population. The second author was a PhD psychiatric nurse, and psychotherapist with long experiences of dealing with migrated people. The third author was a Senior University Teacher with over 40 years’ experience of intercultural education. The fourth author was an Associate Professor in public health specialized in qualitative studies and from another culture. The inclusion of mothers with different backgrounds and who were living in different social settings increased the credibility of the study. The findings were reinforced by word verbatim quotations from informants.

## Results

The qualitative content analysis resulted in the identification of the following four main themes:

- Theme 1—**PPD** with two sub-themes: Psychosomatic symptoms, Burden of responsibility due to feeling of loneliness
- Theme 2—**Mistrust of social services** with two sub-themes: Afraid of losing their children and lack of understanding from the social service.
- Theme 3—**Inadequate healthcare** with two sub-themes: Limited healthcare literacy for migrants’ mothers and Language barrier
- Theme 4—**Women’s coping strategy for well-being** with two sub-themes: Better awareness and understanding of the Swedish system and society, and Freedom and independence in the new country (Table II).

**Table II.** Depression postpartum and life experiences of mothers with immigrant’s background in a region of Sweden.

Categories^1^	Themes^2^	Subthemes	Quotations
Pain, feel bad, stress, lonely, exhausted, too many duties simultaneously, afraid, anxiety, sad, no energy, heavy trauma before arrival in Sweden	Depression/PPD	Psychosomatic symptoms	Pain in the whole body and in my head (M1) Do not feel well, stress, HTA (M2) Feel extremely bad, was at the psychiatry sleeping problems, under medication for depression, anxiety (M3)
Stress, lack of support, lack of support and help from husbands Difficulties in life, feeling of isolation, unemployed, lack of understanding of the Swedish system Continuing to take care of children and home and study despite illness		Burden of responsibility due to feelings of loneliness	Feel Stress (M4) Was devastated, cry, I was a great risk (M3) Stress, feel bad, heavy responsibilities to be single mother, no one understand me (M6) Stress, high education, disappointed (M7) Cannot sleep, feel tired (M8) Feel tired, loneliness, hard, feel dependant of her husband, feel sad (M9)
Discouraged to ask for help from social services, lack of continuity, Afraid to be judged as bad mothers Children will be taken Do not trust the social service, lack of understanding from the social service about their culture and habits, lack of information	Mistrust of social services	Afraid of losing their children and Lack of understanding from social service	Many are afraid. I myself was afraid that they would take my children (M6) They said it is good for your body to walk, it took an hour to reach there” (M4)
Lack of support from healthcare personnel, feeling of rejection, not accepted Inappropriate support from healthcare Lack of continuity in healthcare personnel	Inadequate healthcare	Limited healthcare literacy for migrants mothers	I do not know if they are scare or if they do not understand, you need to see that this nurse have time for you, they must show that I am here to help you, what they show is I am doing a job, that is why people do not open up (M3) It was a new midwife every time (M4)
Language barrier, lack of information, misunderstanding		Language barrier	I have a disease [but] did not know the word in English, I took the medicine that I cannot stand and got really sick lay low for a long time (M6)
Feel happy, satisfy, good care, family support, friend support, husband support, knowledge on law and regulations in Sweden is important, more information, curiosity, open-minded, available care centre, integration, education, good Swedish language	Women’s coping strategy for wellbeing	Better awareness and understanding of the Swedish context and society Freedom and Independence in the new country	Work, education, self-employed, Why could not I have had this information early—I have lost many years (M8) I would like to do something positive, participation in NGO organization (M7) I was born here as a human being., I received education, I felt good, I have become someone else, I received very good support here, Now I ask them all the time—what should I do? (M6) I felt like a real person. When I got mail, my first letter ever, I thought it was so special that I had gotten a letter with my name on it. Also, when I took my children to the dentist they really approached me well. O my God I was so very happy. (M9)

### Postpartum depression

#### Psychosomatic symptoms

All the mothers reported symptoms of depression and described the symptoms as tiredness, stress, sleeping problems, pain, sadness and anxiety. One woman reported that she wanted to take her life both while coming to Sweden and after giving birth in Sweden. Although the mothers expressed depression after childbirth in their narratives, they did not directly associate their symptoms to depression. One woman expressed “*Pain in the whole body and in my head”’ (M7, primary school, 2 children*) and another reported that she “*Did not feel well, had stress and hypertension” (M2, primary school, 2 children).*

Another woman stressed that she *“felt very bad, anxious, had sleeping problems and therefore was at the psychiatry unit and had medication for depression” (M4, primary school, 3 children).*

One woman revealed that she “*was devastated, cried, and was at a great risk of suicide” (M3, secondary school, 2 children).*

Another woman said that she “*could not sleep and felt tired” (M5, university, 4 children)*. Some women experienced trauma in their home countries before moving to Sweden. A woman was treated badly in her home country because of giving birth while still being young and single. She stressed that *“It was hard, and I was thorne and I was scared” (M3, secondary school, 2 children)*. Another woman reported difficulties in child delivery in her home country. Most of the mothers were younger when having their first pregnancy and child.

#### Burden of responsibilities due to feeling of loneliness

All women confessed to being exhausted because of the heavy load of responsibilities at home, having to take care of the children, household and husband.

Some women felt ashamed because of complaining about their social situation “I *am tired all day and night, the baby is crying, but I feel ashamed that I complain as I have a wonderful daughter” (M1, university, 1 child*).

Several of the women pointed out that they mix up their overwhelmed life situation with the stress of having a new baby.

They were alone taking care of the household since the husband was working and they had to take care of the children without help from their family or husband. *“The first time in Sweden was awful, it was winter, I was alone with my husband, and he went to work each morning” (M6, university, 2 children)*.

Some women expressed their loneliness and dependency and stressed that it was “*hard to feel dependent on their husband as she had not been that in the home country” (M1, university, 1 child).*

Loneliness was a recurrent feeling among most of the women. A woman said that it was a *“heavy responsibility to be a single mother” (M4 primary school,3 children)*. They also said that it affected their health being so lonely in Sweden. One woman mentioned loss of identity and independence. Another woman said that her husband told her that loneliness is common in Sweden. A woman revealed that she tried to overcome her stress and feeling of non-belonging by taking her baby out in nature and walking around a lot and that this activity helped her significantly.

All of the women in the study mentioned the frustration of not having a job, while several of them had worked in their country of origin. This situation made several women frustrated. *“I would like to do something” (M4, primary school, 2 children)*. One woman said that having a job is one of the most important presumptions *“for their self-esteem, to have a job, earn money and take part in the society” (M9, primary school 6 children)*.

Another woman stressed that *“It was like torture for me not having a job. I have an education, being unemployed was tough for me” (M8 university 2 children)*.

### Mistrust of the Swedish social service

#### Afraid of losing their children

Several mothers reported that they were informed by their fellow citizens about how they should behave when in contact with the Swedish authorities. They were told not to tell the authorities about problems, because this could lead to judging them as “bad mothers,” taking their children. *“Many are afraid. I myself was afraid that they would take my children” (M6, university, 2 children)*. One of the mothers was told that it was very bad if the child was dirty in pre-school; so, she gave a shower to the child every morning. The child got eczema and when she went to the doctor with the child, he told her that she must reduce the frequency of giving shower to the child. “*They take the child if the child has dirty clothes, poor child I showered her every morning with shampoo it was cold she got eczema” (M10, university, 3 children)*. One of the mothers said that the Social Service had a meeting with her and her husband as one of the daughters was angry at them as parents. In this case, the Social Service was helpful both to the parents and for the daughters “So *now they do not consider talking with the Social Service as they know that if the children have their rights, the parents also have theirs” (M9, primary school, 6 children)*.

#### Lack of understanding on the part of the Swedish social services

They revealed that they were afraid that the Social Service would not understand their culture and habits and therefore might take their children. They also stressed how challenging it was to find a balance between the Swedish system and their own culture on how to raise children. One woman described that her fellow citizens were struggling to maintain and keep their culture/tradition and had little interest in integrating in Swedish society and culture. *“They are afraid, they do not want to integrate, so they are closed… only their culture is important, and they want to keep it alive” (M6, university, 2 children)*.The migrant mothers didn’t ask for help, from outside their inner family, to raise their children as the normality in their culture is to encounter difficulties in everyday life and with children, they did not want to open their life to others. *“Asking for help in Sweden is good but asking for help in many cultures is bad” (M7 primary school, 2 children)*.

A woman stressed that Swedish social services needed to construct a different and more continuing way of informing migrants about childbirth, upbringing issues, Swedish culture, society, healthcare systems, welfare programs etc. Another woman pointed out that different meeting places, family centres, and local gatherings must be planned by the Swedish social services to prevent a lot of mistakes and guide mothers and fathers in the Swedish system to function in a new country. She said that *“To receive information about the Swedish system and care was very important, especially when you have just given birth to a new little person. I wish for parent education, not to wait several years” (M6 university, two children)*.

One mother was worried about her son not having a good time in preschool as he refused to go ther*e*, the mother was ignored by the teacher and by the social service. *“Then I refused to let him go to the preschool he was home with me for four months” (M4, primary school, 3 children)*. The mother felt that no one was listening, not the personnel at pre-school, not the social services representative, and the only thing she could do was to protect the child by having him at home. Then she did not get her financial contribution from the social service.

Some of the mothers felt that they had a hard time taking care of the children and being at Swedish for immigrants’ courses and taking care of the family. The mothers having lower education expressed that the husbands were not taking care of the household. *“The two I have experienced with I give zero points, laugh” (M7 primary school 2 children)*. And for the single mothers the burden was heavy – *“So I was really busy learning the language, I forgot about my pregnancy, and I got sick” (M2, primary school 2 children)*. Some mothers said that they get very stressed as they have children going to different places and it takes them a very long time in the morning and after Swedish for Immigrants (SFI) to come home. The social service had little understanding of the mothers’ burden. “*They said it is good for your body to walk, it took an hour to get there” (M4, primary school, 3 children).*

### Inadequate healthcare

#### Limited healthcare literacy for migrant mothers

Several mothers had experienced a lack of continuity, among staff in healthcare “It *was never the same midwife, each time a new one” (M4, primary school, 3 children)*. *They* mentioned that the lack of continuity made it difficult to build trust and it was difficult for them to rely on them and “open-up”. Several women reported that authorities and healthcare, sometimes being ignorant of their needs. One mother gave birth to a child with heart problems, and she said that the hospital personnel took the child away without telling her about the child’s condition and how this caused a lot of stress for her. The same mother talked about another time becoming so happy when having a welcoming and kind word from a nurse asking about her health situation, and how good she felt with someone showing positive interest in her. *“They need to show I am here for you; I am here to help. One nurse, I really like her, asked me how are you feeling and I became so much happier” (M3, secondary school, 2 children).*

The women who experienced non-attendance and unsuitable care, they did not feel that they were “welcome” and accepted as patients.: *“I don´t know if they are scared or if they don´t understand, “you need to see that the nurse has time for you and that ’’ they must show that I am here to help you, not just doing her job” (M3, secondary school, 2 children)*. Some women revealed that they did not get medical help because of not knowing where to turn to if needed. *“The baby was crying all the time, and I needed to know that she was ok, but it was really hard to have an appointment” (M1, university, 1 child)*. She also said that it is important to understand that, only because you are in the system, having a residence permit and social security number does not mean that you understand the system “*Because being in the system does mean knowing the system” (M1, university, 1 child)*. Almost none of the mothers had completed the Edinburgh Postnatal Depression Scale (EPDS) screening or been asked about their own missed well-being. They did not have questions about their well-being “*I have not filled in any questionnaire and no one asked about how I felt” (M10, secondary school, 3 children)*.

#### Language barrier

Several mothers described that the language was a big obstacle with meeting with the health care. They had numerous stories about misunderstanding that affected the quality of the health care and in some cases the health care had either not been provided or the woman had been mistreated. *“The doctor was late, and the interpreter had to go to another meeting, there was no examination, I did not feel well but the doctor said the child is not fixed, you can go home” (M4, primary school, 3 children)*. The mother did not get a medical investigation and had the baby in the ambulance later that night several weeks too early.

One mother told me that she became mistreated because she did not know the name of her disease. *“I have a special disease, but I was still given medicine that I cannot eat. Even though I said this, the midwife said that I must take the medicine. I became very ill, and I was in hospital, and it took several months to recover” (M6, university, 2 children)*.

To be in an educational program and able to master the Swedish language were reported by several mothers being the most important factor*s* to be integrated in the society The women were in critical situation till the time they underwent SFI (language education) and having information about Swedish system *“Why could not I get this information in the beginning—I lost many years” (M 6, university, 2 children)*. *“I wanted to know the language, but I did not know the language. I did not find the person to ask” (M1, university, 1 child*).

### Women’s coping strategies for well-being

#### Better awareness and understanding of the Swedish system and society

All mothers were convinced that information and education are the ways to get integrated in Swedish society. One mother stressed that *“the best thing for migrants is to really get information about rituals and laws in Sweden, how you raise your children, how to behave against your family, I know a lot of people not knowing about the Swedish system and because of that families got confused and splitted up” (M2, primary school,2 children).*

Mothers also reported that good support from the social service and health care is of importance. A woman expressed her satisfaction by saying that “I *was born here as a human bein*g, *I received education, I felt good, I have become someone else, I received very good support here. Now I ask them all the time for advice about what I should do (M6, university, 2 children)*. Another woman revealed that now that she knows more about the Swedish system everything is significantly easier and that her proud father said jokingly, *“Pity that you have become a Swedish girl” (M2, primary school,2 children). The* same woman reported that she has got help and goes regularly to a health clinic and a psychologist for counselling sessions when needed.

#### Freedom and independence in the new country

Most women described both having a hard time during their first time in Sweden and expressing gratitude to be there as well. One said, *“It feels that I only said bad things, but I think Sweden is a very good country to be a parent in” (M9, primary school, 6 children).*

Especially mothers from east Asia who expressed that they had freedom and independence here. *“I felt like a real human being when I got my first letter in my one name”* (*M4 primary school, 3 children)*. Another mother said, *“Before I came to Sweden, I was a simple woman and I thought that what others said was right, but now I am not thinking that others always have right” (M2, primary School, 2 children).*

One mother talked about starting group meetings for people from different countries, with help from the Red Cross. *“I wanted to do something positive” (M8, university, 2 children)*.

Another mother living in an area having several migrants also reported about starting a parental group dealing with issues and different information concerning parenthood in Sweden. *“We talked about that we had to change some attitudes and not being afraid of Sweden”* (*M6, university, 2 children)*.

One mother talked about curing her stress and feeling of loneliness by taking her two children outside walking in the close forest. *“When I came to Sweden I took my children outside, even to the forest and I felt better” (M10 secondary school, 3 children)*.

Several women reported that they had support from their husbands. *“He helps me, even if he does not agree with me directly, he stays at home with the kids when I go out. He says go out now and meet people” (M6, university, 2 children).*

## Discussion

This study aimed to investigate migrated mothers’ life experiences of motherhood and PPD. The result revealed that PPD was common among the mothers, if we start from the symptoms that the women described (American Psychiatric Association, 2013). The results from this study is in line with previous research showing that PPD and depression are common among immigrant women (Alhasanat & Fry McComish, 2015; Halbreich & Karkun, 2006; Van Lieshout et al., 2011). The mothers themselves did not use words like depression or mental illness. They described their mental illness in terms of somatic symptoms, such as pain in the body, headaches, sleeping problems, and feeling bad. It seemed as if depression for the migrant mothers became psychosomatic and could only be expressed as a somatic feeling. This has also been found in other studies (Dennis & Chung Lee, 2006; Tobin et al., 2015). This phenomenon has its origin in the mother’s habit of talking about mental illness due to their social norms and culture. For the mothers it was a matter of trust to reveal their mental status and it took time for them to find words describing the same. For numerous migrants, depression and other mental illnesses are associated with madness (Dennis & Chung Lee, 2006; Schmied et al., 2017) and this also made it difficult to open up and talk about themselves. These results give an indication that healthcare staff should be aware that somatic pain without any somatic explanation among migrant women could be a sign of depression. Examining the woman’s mood and social situation increases the possibilities of identifying migrant women with PPD.

It is important that health care staff possess knowledge about immigrant women’s numerous risk factors for developing depression. Immigrant mothers are more frequently exposed to PPD than non-immigrants due to pre-migration trauma and stress (Collins et al., 2011; Fung & Dennis, 2010; Van Lieshout et al., 2011). It is especially important to identify migrant mothers’ depression as they have the responsibility of the children and as do not have any social network that could compensate for the mother’s depression. PPD is especially serious during the first year due to the mother's inability to interact with the child and this has a negative impact on the cognitive development of the baby. This can also negatively affect attachment which in turn affects later relationships for the child (Agnafors et al., 2013; Deave et al., 2008; Netsi et al., 2018)

All women in the study exhibited a burden of social and household responsibilities because of tradition/culture. The women with low education were engaged with “the women’s duties to take care of children and household” whereas those with higher education had help from their husbands but still they oversaw the children and the household. Gender norms and divisions in households have been underlined in other studies (Cislaghi, 2018). Above all, being in a new country added to their feeling of being exhausted which made several mothers very vulnerable when partners or husbands were not involved in taking care of the children or household chores (Cislaghi et al., 2018). As the mothers used to receive social support from the extended family and friends in their country of origin, they expressed loneliness as they had no social network in Sweden. Loneliness, poor economy and difficulties getting a job are all risk factors for developing PPD (Docherty et al., 2022). It is well-documented that immigrant women may develop PPD because they are exposed to the usual stresses of motherhood, but also with cultural and language barriers, social isolation, discrimination, and financial problems (Collins et al., 2011; Fung & Dennis, 2010).

Some of the mothers expressed mistrust of social service. The lack of confidence in the social service may have affected the mother’s willingness to ask for help or tell the social service about their struggles and difficulties in being a parent in Sweden. Studies have found that fear of the social services is correlated to a feeling of powerlessness in the new country (Deng & Marlowe, 2013; Osman et al., 2016). But studies have also shown that social services in Sweden are twice as likely to take children from immigrant families into care, Vård- och omsorgs analyse study 2018; SAVE study (Persdotter & Andersson, 2020). Mothers were not aware that social services may help as well in their parenting role. The lack of confidence in social service and suspicion might have contributed to the woman’s mental problems as they were afraid of asking for help. Nevertheless, their trust in social service increases with time in Sweden. Some mothers could after a while assess whether claims from fellow citizens were true or not. The women who had contact with the family centre and the integration project expressed that they gained trust in the staff and were able to ask them about the mission of the social service. It seems crucial that information about the social services to the immigrant parents is an important task. It is especially important to inform migrant parents about children’s rights and parents’ obligations, and that voluntary support is offered by social service to prevent children being taken to care in case of behaviour problems (Persdotter & Andersson, 2020). In order to create good conditions for the mothers to understand society’s demands and support system, it takes time to provide information and to build trust among immigrants. It is also important that the fathers have the same information as they can either support or stop the women from taking part in society. The family centre and the integration project have these qualities of building trust as they have better skills and cultural understanding about immigrant women and their needs. Several international studies have pointed out immigrant parents’ daily stressors to adjusting as a parent in a new country (Deng & Marlowe, 2013; Lewig et al., 2010) and obstacles they face as parents owing to the lack of information about parenting systems and the rights in the host country.

The Swedish healthcare system is organized to give equal care to all citizens. Due to digitalization, it is important to read, understand value information and to be able to communicate health messages, this is called “health literacy”. Health literacy is a concept not solely reliant on individual capabilities but also on organizations’ ability to make health-related information and services equitably accessible and comprehensible (Santana et al., 2021). For immigrants, health literacy in Sweden can be difficult to access as they may have limited knowledge on digitalization, language and knowledge about where to find the information (Olofsson, 2022; Wångdahl et al., 2017). The mothers had difficulties understanding and accessing the Swedish healthcare system. Moreover, they felt that even when their health was a problem it was not properly addressed. When healthcare staff do not have the opportunity to provide sufficient good care due to a lack of time or resources, there is a risk that the patients would be negatively affected (Skoog et al., 2022). It was reported previously that healthcare systems are complicated bureaucracies and that even highly motivated and educated individuals may find them too confusing, especially when persons are more vulnerable due to poor health (Santana et al., 2021). An explanation for inadequate care is lack of cultural knowledge among the Swedish health care staff (Berlin et al., 2006 (Fung & Dennis, 2010). Few of the mothers had been offered EPDS-screening and when asked questions, they either did not understand the questions, or they did not answer truthfully. A Swedish register study found that those born outside Sweden and reported poor self-rated health were at increased risk of not being offered screening for PPD (Bränn et al., 2021). As these women are particularly vulnerable, it is of great importance to investigate why the EPDS may miss this group most in need of being identified. Research is divided in the view of whether EPDS is sufficiently well evaluated for women outside the Western world, with other researchers believing that it can be used but the cut-off value may need to be adjusted (Collins et al., 2011 (Dennis et al., 2017). A recent study conducted for Swedish Healthcare Nurses found that the nurses were frustrated over the difficulties of EPDS screening in their daily work (Skoog et al., 2022). The nurses said that they were afraid of missing mothers with signs of PPD and felt frustrated in handling communication associated with the translated version of the E P D S and cultural implications of PPD (Skoog et al., 2022). Another study found that lack of time can be why the healthcare staff is not able to carry out the EPDS screening (Skoog 2017). It seems like the healthcare system does not adjust according to the patients’ needs and the time for the healthcare staff to talk and be familiar with the mothers have decreased.

Swedish healthcare literacy needs to improve the migrants’ women understanding of health information and services by focusing on the migrants-provider communication and quality digital tools. To provide accurate health information and services that migrant women can easily find, understand, and use to inform their decisions and actions is required from the Swedish healthcare system as described in a previous report (US. Department of Health and Human Services, healthy people 2020).

We found that mothers complained that they met different healthcare personnel, during their pregnancy and after delivery. It has been indicated previously that migrant women required support through appropriate services, and continuity in the care to fully understand their situation, especially as they often are the family supporter and that they are important for children’s well-being and development (Deave et al., 2008; Schmied et al., 2017). The current organization in hospitals and primary care does not seem to meet the needs of migrated mothers except the mother being in contact with the family centre. Surveys among the Swedish population underlines that Swedish healthcare from the patients’ perspective is difficult to access and not always available for them as well as it suffers from lack of continuity. In the accurate health system, it is complicated to get an appointment and that the patients are constantly confronted with different health staff (Styrning och vardkonsumtion ur ett jamlikhetsperspektiv, 2018). It is important that maternity care is organized to support immigrant mothers’ special needs. Continuity has been shown in several research studies to have positive health effects for the child and the mother as well and affect the child’s development positively (Axelsson et al., 2016).

A recent study found that immigrant and refugee women often had access to the same services as women born in the host country. However, there were differences as migrant women had more challenges including accessing services, cultural differences, language barriers, limited health literacy, insufficient support, transport issues and limited financial capacity (Rogers et al., 2020). In the same study, the conclusion was that migrant mothers needed a healthcare system organized to give effective communication, psychosocial and practical support, support to navigate systems, flexible and accessible services (Rogers et al., 2020).

Language barrier was a limitation, making it difficult to communicate with health personnel and to get appropriate care probably because of lack of mutual understanding and communication. Research indicates that language is a barrier and obstacles to reach proper health care for immigrant women (Schmied et al., 2017). The mothers in the study also had experiences where they had been maltreated probably due to language limitation and the limited time of the healthcare staff. Migrant mothers need specific arrangements, and the healthcare staff need time to prepare for the mothers along with a translator. To give EPDS screening in the mother’s language is more time consuming. It also takes more time to give the mothers time to adjust to questions about themselves and their mental health. Studies of healthcare nurses’ work situation found that child health nurses were dissatisfied with the work situation, with a very tight schedule and limited visits to the immigrant mothers. This held true for both nurses and midwives (Skoog 2022; Axelsson et al., 2016).

The mothers in this study had different challenges to be a part of Swedish society. To be a migrant mother without work is a problem for society and the integration. Ahlbertz (2012) found in her study that the government constructs the non-work of immigrant women as “a competence problem”, a “family problem,” and a “participant problem.” The government solutions are also formulated based on these constructs. Ahlbertz’s (2012) conclusion is that “increasing competence” is not the answer for migrated women to be employed. What is required is a redefinition of what “is competence,” and the government needs to reflect on the labour market’s demands to help the immigrants adapt to that. Despite the fact that mothers experienced difficulties and poor mental health, and their tireless work to become employable, they have shown a strong ability to overcome their difficulties in their own way.

### Clinical implications

This study revealed that migrant mothers need Social Service and Healthcare being adapted to their specific needs. The following provisions are needed:

- Investigating how to improve EPDS screening in Child Health Services for migrant mothers.
- Providing all immigrant parents education on what it means to be a parent in Sweden.
- Instilling intercultural competence, continuity in care, and multidisciplinary knowledge among Child Health Care staff working with immigrant parents
- Facilitating cohesive activities (by Social Services) for migrant parents wherein they are offered an integration program—including community knowledge, parenting knowledge, and language training and practice.

Further research is needed to evaluate the impact of migrant mothers’ depression on children.
